# Mild hypothermia during advanced life support: a preliminary study in out-of-hospital cardiac arrest

**DOI:** 10.1186/cc6809

**Published:** 2008-02-29

**Authors:** Cédric Bruel, Jean-Jacques Parienti, William Marie, Xavier Arrot, Cédric Daubin, Damien Du Cheyron, Massimo Massetti, Pierre Charbonneau

**Affiliations:** 1 Medical Intensive Care Unit, Caen University Hospital, Avenue côte de Nacre, 14033 Caen cedex, France; 2 Department of Biostatistics and Clinical Research, Caen University Hospital, Avenue côte de Nacre, 14033 Caen cedex, France; 3 Department of Emergency Medical Services, Caen University Hospital, Avenue côte de Nacre, 14033 Caen cedex, France; 4 Department of Thoracic and Cardiovascular Surgery, Caen University Hospital, Avenue côte de Nacre, 14033 Caen cedex, France

## Abstract

**Introduction:**

Induction of mild hypothermia after cardiac arrest may confer neuroprotection. We assessed the feasibility, safety and effectiveness of therapeutic infusion of 2 l of normal saline at 4°C before return of spontaneous circulation during cardiopulmonary resuscitation after out of hospital cardiac arrest.

**Methods:**

This was a prospective, observational, multicenter clinical trial conducted in Emergency Medical Services units and in a medical intensive care unit at Caen University Hospital, Cen, France.

**Results:**

In patients who had suffered out of hospital cardiac arrest, hypothermia was induced by infusing 2 l of 4°C NaCl 0.9% over 30 minutes during advanced life support prior to arrival at the hospital. A total of 33 patients were included in the study. Eight patients presented with ventricular fibrillation as the initial cardiac rhythm. Mild hypothermia was achieved after a median of 16 minutes (interquartile range 11.5 to 25.0 minutes) after return of spontaneous circulation. After intravenous cooling, the temperature decreased by 2.1°C (*P *< 0.0001) to a mean body temperature of 33.3°C (interquartile range 32.3 to 34.3°C). The only observed adverse event was pulmonary oedema, which occurred in one patient.

**Conclusion:**

We concluded that prehospital induction of therapeutic hypothermia using infusion of 2 l of 4°C normal saline during advanced life support was feasible, effective and safe. Larger studies are required to assess the impact that this early cooling has on neurological outcomes after cardiac arrest.

## Introduction

Out of hospital cardiac arrest (OHCA) is common and associated with a poor outcome, despite improvements in cardiopulmonary resuscitation (CPR) [1,2]. Two major complications may occur after resuscitation from cardiac arrest. The first is postresuscitation disease, which occurs between 4 and 24 hours after resuscitation and is characterized by an ischaemia/reperfusion syndrome and a systemic inflammatory response syndrome [3]. The second complication is severe anoxic brain injury, which kills two-thirds of survivors [3,4].

In a hospital setting, mild hypothermia induced 4 to 8 hours after OHCA improves the outcomes of comatose survivors in whom ventricular fibrillation (VF) is the initial rhythm [5,6], and this strategy is currently recommended by the International Liaison Committee On Resuscitation [7]. Using external or endovascular cooling devices, it takes 219 to 480 minutes to achieve cooling to 33°C [6,8,9]. Infusion of cold crystalloids after hospital admission effectively lowers body temperature in resuscitated patients [10,11]. However, studies of animal models of cardiac arrest suggest that the timing of induced hypothermia is a major determinant of survival [12,13]. In an animal model early cooling resulted in significantly better neurological outcomes than did delayed cooling after return of spontaneous circulation (ROSC) or normothermic resuscitation [12]. In addition, a recent case report conducted by Bernard and coworkers [14] suggested that inducing cooling before ROSC may be advantageous.

In the present study we aimed to determine the feasibility, safety and effectiveness of cooling patients before ROSC during advanced life support (ALS) in the field before their arrival at the emergency department.

## Materials and methods

### Study protocol

This prospective, observational, clinical trial was conducted between October 2004 and October 2006 and involved both Emergency Medical Services (EMS) and the medical intensive care unit (ICU) at the University Hospital in Caen, France. This study was supported by an academic grant (registration number 2004-R-58) and was approved by the Institutional Ethics Committee of Caen University Hospital, in accordance with the European Guidelines for Good Clinical Practice. In accordance with French law, the next of kin were orally informed about the trial by the prehospital emergency team at the time of inclusion, and written consent was systematically obtained from participants' next of kin within 24 hours. Furthermore, written consent was obtained from all survivors.

### Study design

Consecutive comatose patients aged 18 to 70 years suffering nontraumatic OHCA were eligible, regardless of their initial cardiac rhythm. Patients with the following were excluded: pregnancy; response to verbal commands after the ROSC; traumatic aetiology of cardiac arrest; and enrollment in another study.

Patients were treated in accordance with current European Resuscitation Council guidelines [15]. After ROSC, patients were ventilated manually and ECG (Lifepak^®^12; Medtronic PhysioControl, Redmond, WA, USA), pulsatile oxygen saturation (Oximax^®^; Nellcor, Pleasantan, CA, USA) and end-tidal carbon dioxide (FilterLine^® ^H Set; Medtronic PhysioControl) were monitored continuously. The arterial blood pressure was measured noninvasively every 5 minutes and the oesophageal temperature was monitored continuously using the Data Therm™ monitor for adults (Medical Geratherm AG, Geschwenda, Germany). EMS physicians placed oesophageal temperature probes after tracheal intubation in all patients.

**Figure 1:**
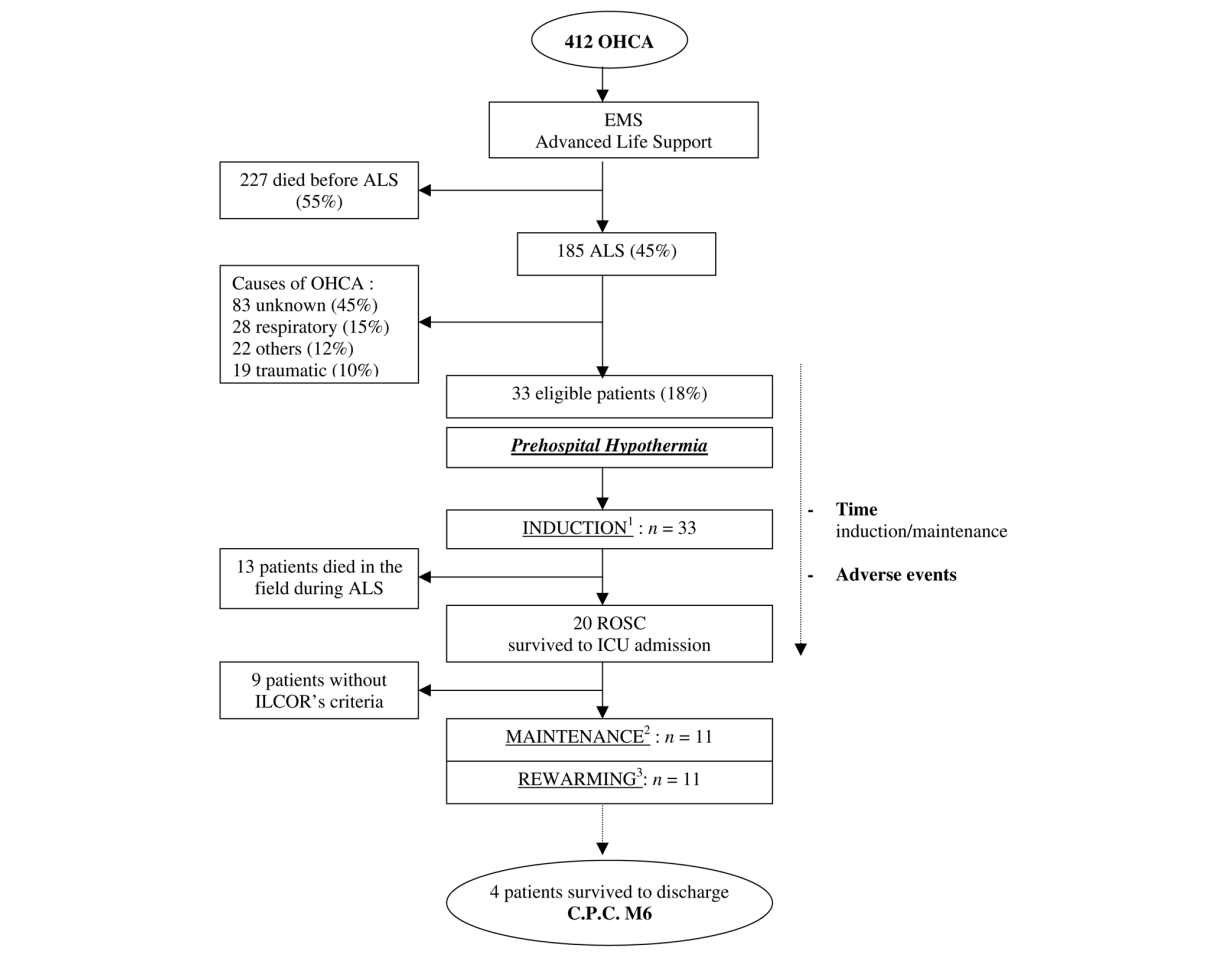
Study design and protocol. ^1^Induction: hypothermia was induced by infusion via peripheral intravenous line of 2000 ml of NaCl 0.9% at 4°C. ^2^Maintenance hypothermia was achieved by endovascular cooling using Coolgard 3000™, with a target core temperature of 33°C over 24 hours. ^3^Rewarming was by endovascular warming by Coolgard 3000™ from 33°C to 37°C over 24 hours. ALS, advanced life support; EMS, Emergency Medical Services; ICU, intensive care unit; ILCOR, International Liaison Committee On Resuscitation; OHCA, out of hospital cardiac arrest; ROSC, return of spontaneous circulation. CPC M6: Neurological status was evaluated at 6 months using the Pittsburgh Cerebral Performance Category (CPC)

Cooling was performed as soon as possible during ALS by EMS personnel. The EMS team was composed of one physician and two paramedics, who did not require special training. Specifically, hypothermia was induced during ALS at the arrival of EMS by infusing 2 l of NaCl 0.9% at 4°C over 30 minutes. The ice-cold normal saline was infused through a peripheral intravenous line that was 18 gauge or larger, using a pressure bag inflated to 300 mmHg. All patients were sedated after ROSC by ketamine hydrochloride (1 mg/kg per hour) with fentanyl (1.5 μg/kg per hour) for sedation and received atracurium (0.5 mg/kg per hour) to prevent shivering. The clinical trial required equipment to be added to the EMS units. One-litre bags of normal saline were stored in a refrigerator at 4°C before use, and each EMS unit was equipped with refrigerators capable of storing several 1 l bags of normal saline at 4°C. In the hospital, hypothermia was continued for 24 hours using an endovascular cooling catheter (Icy™ Catheter; Alsius, Irvine, CA, USA), which was inserted into the inferior vena cava via the right or left femoral vein and connected to a cooling device (Coolgard 3000™; Alsius) in the ICU. The oesophageal temperature was recorded online and the target temperature was 33 ± 1°C. Patients were then rewarmed within 24 hours to 37°C. The study is summarized in Figure 1.

### Data collection

In addition to baseline resuscitation characteristics, including both clinical and biological data, we monitored body temperature at the following times: before the induction of mild hypothermia (step 0), after the rapid infusion of normal saline at 4°C (step 1), upon arrival in the emergency room (step 2), in the ICU (step 3), and after 30 minutes (step 4) and 12 hours (step 5) of use of the endovascular cooling device. EMS personnel also recorded temporal data: the no-flow interval (time between patient collapse and initiation of CPR), the low-flow interval (time from beginning of CPR to ROSC), and the time from the ROSC to target temperature (<34°C). Baseline clinical and laboratory data recorded in the emergency room included blood gas analysis, blood levels of sodium, potassium, chloride, calcium, serum albumin, serum urea nitrogen, serum creatinine, haematocrit, haemoglobin, white blood cell count and platelet count. Adverse events, including bleeding, arrhythmia (ventricular fibrillation or tachycardia), pulmonary oedema and electrolyte disturbances, were also recorded.

Neurological outcome at 6 months was assessed using the five-grade Glasgow-Pittsburgh Cerebral Performance Categories (CPC) [16]. A favorable neurological outcome was defined as a CPC of 1 (good cerebral performance) or 2 (moderate cerebral disability) on a five-category scale; the other categories were 3 (severe cerebral disability), 4 (coma or vegetative state) and 5 (death). Patients with good recovery or moderate disability had sufficient cerebral function to live independently and work at least part time.

### Statistical analysis

Quantitative variables were expressed as mean ± standard deviation or median (interquartile range), as appropriate. Qualitative variables were expressed as numbers and percentages. All consecutive patients were included in the assessment of the effectiveness of lowering body temperature (between steps 0 and 1), in accordance with the intent-to-treat principle. In order to modelize body temperature with time (steps 0 to 5), we used the concept of mixed models with random intercepts to account for repeated measurements within the survivor group. All statistical analyses were performed using Epi-Info V6 (Centers for Disease Control and Prevention, Atlanta, GA, USA) and SAS V9.1.3 (SAS Institute Inc., Cary, NC, USA). *P *< 0.05 was considered statistically significant.

## Results

**Figure 2:**
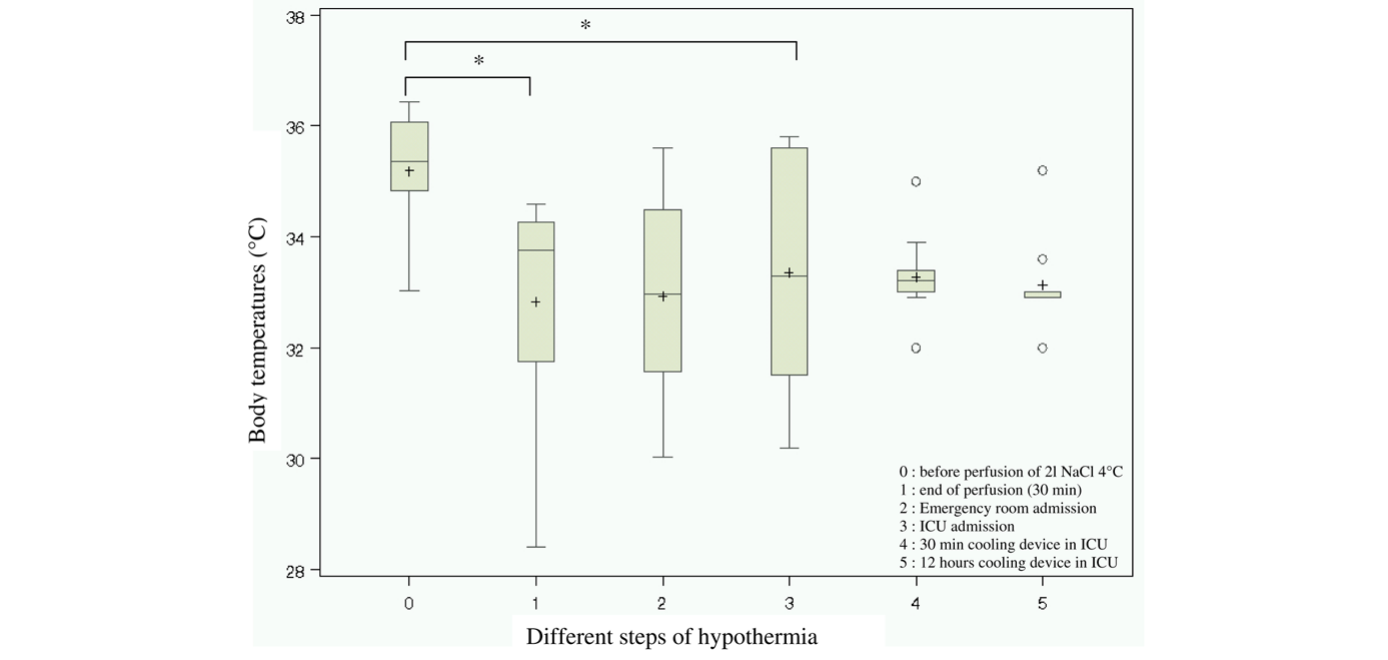
Boxplots of body temperatures. Figure 2 contains six boxplots for 11 patients who received endovascular cooling. On the x-axis and for each boxplot, the number corresponds to the differents steps; the y-axis shows body temperatures. The shaded box denotes the middle 50% of the data, and hence the lower and upper ends of the box denote the 25th and 75th percentiles, respectively. The solid black horizontal line through each shaded box denotes the median of the distribution and the black cross denotes the mean. The vertical solid black lines ('whiskers') reach out to 1.5× the interquartile range. Circles above the whiskers denote individual extreme observations. Step 0: temperature before infusion of 2 l of NaCl 0.9% at 4°C; step 1: temperature at the end of perfusion; step 2: initial temperature in emergency room; step 3: initial temperature in ICU; step 4: temperature after 30 minutes of endovascular cooling (Coolgard 3000™); and step 5: temperatures after 12 hours of endovascular cooling (Coolgard 3000™). *P < 0.05.

Thirty-three patients were included in this study (Figure 1). The baseline characteristics of the patients are shown in Table 1. In the 33 patients, the mean temperature decreased significantly (*P *< 0.0001) by -2.1°C ± 0.29°C after intravenous cooling to a median temperature of 33.3°C (32.3°C to 34.3°C). Twenty of the 33 (60.6%) were successfully resuscitated. The median time to reach mild hypothermia (<34°C) after ROSC was 16 minutes (11.5 minutes to 25.0 minutes). Eleven patients survived and were admitted to the ICU, where the endovascular cooling device was used. Boxplots of patients' body temperatures according to different resuscitation steps are shown in Figure 2. In this subgroup, temperatures before (step 0) and after (step 1) infusion of 4°C normal saline decreased significantly by -2.4°C ± 0.43°C (*P *< 0.0003). The temperatures in the kinetics curve revealed nonsignificantly passive rewarming, with a +0.26°C ± 0.35 of temperature variation (*p *= 0.48) between the end of cold normal saline infusion (step 1) and admission to the ICU (step 3). However, the patients' body temperatures were always significantly lower upon hospital admission (step 2; *P *< 0.004; temperature difference -1.13°C ± 0.28°C) and upon ICU admission (step 3; *P *< 0.02; temperature difference of -0.6°C ± 0.21°C) than their initial temperature before cooling (step 0).

**Table 1:** Baseline characteristics

Parameter	Value
Age (years)	53 (47 to 65)
Men (*n *[%])	26 (78.7)
Witnessed cardiac arrest (*n *[%])	26 (78.7)
Basic life support (*n *[%])	9 (34.6)
Ventricular fibrillation (*n *[%])	8 (24.3)
Asystole plus pulseless electrical activity (*n *[%])	25 (75.7)
Defibrillations (*n*)	1 (0 to 4)
Epinephrin (mg)	15 (4 to 20)
End-tidal carbon dioxide at ROSC (mmHg)	33 (19 to 47)
No-flow time (minutes)	10 (5 to 14)
Low-flow time (minutes)^a^	16 (11.5 to 25)
Time from collapse to ROSC (minutes)^a^	25 (20.2 to 35.7)
Time from ROSC to mild hypothermia (minutes)^a^	16 (11.5 to 25)
PaO_2_/FiO_2_^b^	300 (159 to 403)
pH arterial blood gas^b^	7.16 (7.02 to 7.23)
Lactates (mmol/l)^b^	9.5 (3.7 to 11.3)
Serum creatinine (μmol/l)^b^	147 (94 to 179)
Sodium (mmol/l)^b^	138 (136 to 139)
Potassium (mmol/l)^b^	4.1 (3.8 to 4.5)
Calcemia (mmol/l)^b^	2.1 (2.1 to 2.3)
Chloride (mmol/l)^b^	107 (101 to 109)
Haemoglobin (g/dl)^b^	12.9 (11.9 to 13.4)
Haematocrit (%)^b^	39.1 (36.4 to 40.7)
Platelet count (g/l)^b^	165,500 (88,000 to 208,000)

Data are presented as median and their interquartile range or as numbers (percentages). ^a^Twenty out of 33 patients successfully resuscitated. ^b^Biological data among 11 patients admitted to the intensive care unit. FiO_2_, fraction of inspired oxygen; PaO_2_, arterial partial oxygen tension.

Only one patient developed pulmonary oedema, which was characterized by clinical features as well as decreased pulsatile oxygen saturation; development of oedema prompted interruption of cold saline infusion after 1500 ml. There were no adverse events involving bleeding, and neither did any arrhythmia occur. Moreover, we did not observe any cases of re-arrest during transport to the hospital. There were no clinically relevant changes in the biological data recorded at the hospital (Table 1). Fluid loading did not induce haemodilution; the median haematocrit was 39.1% (36.4% to 40.7%). The pH of the arterial blood gas, 7.16 (7.02 to 7.23), exhibited a lactic acidaemia.

Finally, EMS personnel reported no technical difficulties in implementing the protocol.

At 6 months after OHCA, four patients survived, three with a favorable neurological outcome (CPC ≤ 2). The primary adverse effect was anterograde amnesia. The initial cardiac rhythms of these four patients were VFs in three patients and asystole in one.

## Discussion

This study shows that rapid infusion of 2 l of normal saline at 4°C in the field during ALS after OHCA and before ROSC was feasible, effective and safe in terms of decreasing body temperature before arrival at the hospital. We used a standard dose of 2 l instead of a weight-based dose in order to develop an easily managed protocol for treatment of OHCA patients in the field. With early cooling, we observed an ROSC rate of 60.6%; there were no adverse effects associated with the infusion of 2 l of ice-cold normal saline during ALS, except for oedema, which developed in one patient. The time from witnessed cardiac arrest to initial treatment, as well as delays from OHCA to CPR and to ROSC, were similar to those in previous reports [5,6,10,11]. The no-flow time, which was 10 minutes (5 minutes to 14 minutes), was consistant with the criteria for inclusion in the HACA (Hypothermia After Cardiac Arrest) Study Group trial [6]. The median delay between the OHCA and ROSC (25 minutes [20.2 minutes to 37.5 minutes]) was comparable to that reported by Bernard and coworkers [10] (26 minutes [20.3 minutes to 34.5 minutes]) and Kliegel and colleagues [11] (20 minutes [13 minutes to 29 minutes]).

Inducing mild hypothermia during ALS, as compared with post-ROSC early cooling [17,18], may reduce the duration of cerebral ischaemic injury. In experimental models [12,13,19], the time to induce hypothermia was closely correlated with anoxic neurological injury. In the animal model described by Nozari and coworkers [19], 17 dogs had VF cardiac arrest with a no-flow of 3 minutes, followed by 7 minutes of CPR basic life support and 50 minutes of ALS. The dogs were randomly assiged to two treatment groups: an early hypothermia group (34°C), in which cooling was initiated at 10 minutes of VF during ALS, and a delayed hypothermia group, in which colling was initiated at 20 minutes of VF. In the early hypothermia group, seven out of nine dogs survived to 96 hours, five with good neurological outcome. In contrast, seven of eight dogs in the delayed hypothermia group died within 37 hours with multiple organ failure (*P *= 0.012) [19]. Consequently, a small reduction in the delay between cardiac arrest and therapeutic hypothermia may improve neurological outcome after resuscitation. The present study confirmed the feasibility and effectiveness of cooling with a rapid infusion of cold fluid, as was previously reported [10,11,17,18]. Lowering temperatures by infusion of normal saline at 4°C was more effective in this study than in the other studies [10,11,17,18]; we achieved a mean temperature decrease of 2.1°C ± 0.29°C and a median body temperature of 33.3°C (32.3°C to 34.3°C; step 1). Differences in the cooling protocol could account for this finding. Recently, Kämäräinen and coworkers [20] observed a decrease of 2.5°C (nasopharyngeal temperature) in five patients after paramedics infused 14 ml/kg of Ringer's acetate at 4°C during CPR; four patients died in the field.

Once the target temperature was reached, patients remained within the temperature range of 32°C to 34°C. Hypothermia induced in the field persisted from the end of infusion until admission to the ICU. However, rewarming was reported by Kliegel and colleagues [21], who proposed maintenance of mild hypothermia by repeated cold infusions. The consequences of these thermal deviations for cerebral metabolism remain unknown. Because the cerebral metabolic rate decreases by 6% to 7% for each 1°C decrease in temperature [22], it could be preferable to continue the 'cold chain' without interruption until admission to the ICU.

A major concern about rapidly infusing cold fluid in the field is the possibility of inducing pulmonary oedema in resuscitated patients. In the present study, initiating hypothermia during ALS before ROSC reduced volume infusion during resuscitation and possibly decreased the risk for development of pulmonary oedema. All patients except one received 2 l of cold fluid loading, and the median arterial oxygen tension/fraction of inspired oxygen ratio of patients who survived to ICU admission was 300 (159 to 403). This study confirms the safety of infusing a large volume of cold fluid, consistent with the findings of an echocardiographic study [23] and with those of other studies that tested the safety and effectiveness of this cooling procedure [10,11,17,18]. Kim and coworkers [23] demonstrated that fluid loading with 2 l of normal saline at 4°C had no significant effect on left ventricular function, left atrial filling pressures, pulmonary artery pressure, or central venous pressure. Bernard [10] and Kliegel [11] and their colleagues did not observe pulmonary oedema after infusion of cold Ringer's solution in any of the 22 or 26 patients, respectively, included in their studies. However, these trials were conducted after hospital admission rather than before. In-field cooling was not associated with pulmonary oedema, as initially assessed by chest radiography, in the recent clinical trial conducted by Kim and coworkers [23]; however, only 12 patients out of 63 received the 2 l of cold normal saline after ROSC. Virkkunen and colleagues [18] also found no pulmonary oedema after prehospital infusion of cold Ringer's solution. In addition, we did not observe bleeding complications or metabolic disturbances in any patients included in the present study.

We are aware that this study has limitations. Specifically, there was no control group with which to compare changes in body temperature, renal function, or electrolyte disorders. However, the results of this feasibility study now make it ethically acceptable to conduct a larger, multicentre, randomized trial to assess whether prehospital induced cooling after OHCA confers neurological benefits.

## Conclusion

We conclude that infusion of 2 l of normal saline at 4°C in the field during ALS to induce mild hypothermia in resuscitated OHCA patients is feasible, safe and effective. This approach could represent the earliest and most cost-effective neuroprotective strategy.

## Key messages

• Therapeutic infusion of 2 l of normal saline at 4°C over 30 minutes during resuscitation following cardiac arrest safely and effectively lowers body temperature and can be used as part of ALS after OHCA.

• Inducing mild hypothermia in the field by EMS personnel is reproducible and cost-effective.

## Abbreviations

CPC = Cerebral Performance Categories; CPR = cardiopulmonary resuscitation; EMS = Emergency Medical Services; ICU = intensive care unit; OHCA = out of hospital cardiac arrest; ROSC = return of spontaneous circulation; VF = ventricular fibrillation.

## Competing interests

The authors declare that they have no competing interests.

## Authors' contributions

WM, MM and PC conceived the study. MM and PC obtained the funding. XA was involved in recruiting patients from the EMS at the Caen University Hospital, coordinated recruitment from other centres and was involved in data collection. CB collected data, carried out analyses and drafted the manuscript, which was subsequently reviewed by JJP, WM, XA, CD, DdC, MM and PC. JJP performed the statistical analysis. PC takes overall responsibility for the findings presented here.
